# Impact of inulin and yeast containing synbiotic on calves’ productivity and greenhouse gas production

**DOI:** 10.14202/vetworld.2020.1017-1024

**Published:** 2020-06-02

**Authors:** S. Jonova, A. Ilgaza, M. Zolovs, A. Balins

**Affiliations:** 1 Faculty of Veterinary Medicine, Preclinical Institute, Latvia University of Life Sciences and Technologies, Jelgava, Latvia; 2 Department of Biosystematics, Institute of Life Sciences and Technology, Daugavpils University, Daugavpils, Latvia; 3 Research Laboratory of Biotechnology, Division of Molecular Biology and Microbiology, Latvia University of Life Sciences and Technologies, Jelgava, Latvia

**Keywords:** calves, greenhouse gases, inulin, productivity, *Saccharomyces cerevisiae*, symbiotic

## Abstract

**Aim:**

The research aimed to determine the impact of synbiotic: 6 g of prebiotic inulin and 5 g of probiotic *Saccharomyces cerevisiae* strain 1026 on calves’ productivity and greenhouse gas (GHG) production.

**Materials and Methods:**

The research was conducted with 10 Holstein Friesian and Red Holstein (*Bos taurus* L.) crossbreed calves of mean age 33±6 days and initial body weight 73.4±12.75 kg. We added the synbiotic into the diet of five dairy crossbreed calves (SynG) and five calves in control group (CoG) received non-supplemented diet. The duration of the experiment was 56 days. The weight of calves and amount of methane (CH_4_) and carbon dioxide (CO_2_) in the rumen were determined on day 1, 28, and 56. On day 56, three calves from each group were slaughtered. Meat samples were assessed for some indicators of meat quality. The main methanogens were detected in the rumen fluid and feces.

**Results:**

The weight gain during the whole experiment period of 56 days was higher in the SynG (62.6±13.75 kg) compared to CoG (36.8±7.98 kg) calves (p<0.01). There were no significant differences in the levels of protein (%), fat (unsaturated and saturated – %), and cholesterol (mg/100 g) in meat samples from both groups. At the end of the experiment, the amount of CH_4_ in calves’ rumen in CoG was higher (Me=792.06 mg/m^3^, interquartile range [IQR] 755.06-873.59) compared to SynG (Me=675.41 mg/m^3^, IQR 653.46-700.50) group (p<0.01). The values for CO_2_ were also increased in CoG (Me=4251.28 mg/m^3^, IQR 4045.58-4426.25) compared to SynG (Me=3266.06 mg/m^3^, IQR 1358.98-4584.91) group (p=0.001). There were no significant differences in the calves’ weight and certain methanogen species in rumen liquid and feces on the 56^th^ day of the experiment. Significantly higher results in the parameter total prokaryotes (V3) (bacteria+archaea) in rumen fluid were in SynG, whereas significantly higher results in the parameter total methanogens Met630/803 in rumen fluid were in CoG, p<0.05.

**Conclusion:**

The main results showed that the synbiotic can increase the daily weight gain in calves and decrease the amount of GHG in rumen but does not impact different methanogen species in rumen liquid and feces and meat protein, fat, and cholesterol levels.

## Introduction

The United Nations has calculated that the global demand for food will double by the year 2050 when the population is going to increase up to 9.8 billion. This demand is going to be a great challenge for agricultural industries as the world will need extra food for the growing population. The provided food will have to be healthy, nutritious, and sustainably produced [1]. The greatest challenge will be to reach this goal and at the same time reduce the emission of greenhouse gases (GHGs) from the agricultural sector. The global temperature of our planet has increased by 0.85° since 1880 mainly due to human activity [2]. Livestock products are responsible for increased GHG emissions compared to other food sources. The emissions of dairy cattle are the result of complex biological processes that occur in animal digestive system. The most important is methane (CH_4_), which is produced as a by-product of the digestion processes [1]. The second GHG emitted from dairy cattle that contributes to global warming is carbon dioxide (CO_2_). The global warming potential of CH_4_ is 21 times more than CO_2_ [3]. In general, the CO_2_ produced through respiration processes is not considered as a great source of GHG emissions, since it is assumed that the observed amount of CO_2_ by plants is equivalent to the amount emitted by livestock. Furthermore, the consumed carbon is used in animal tissues and products, such as milk [4].

Nowadays, researchers are working on ways of reducing GHG emissions from all agricultural sectors, including livestock farming and increasing animal productivity. The aim is to produce less CH_4_ per unit of meat or milk [5]. Rumen methanogens use H_2_ and CO_2_ produced by other fermentative members of the ruminal microbiome, to create CH_4_ [6]. This gas not only negatively impacts our surrounding environment but also causes energy loss to animals. It is proven that about 2-12% of the ingested feed energy is lost as CH_4_ [7]. Changes in animal diet and addition of different feed additives have been identified as main ways for the mitigation of CH_4_ production and the improvement of animal health and productivity [1]. The prebiotics or oligosaccharides are non-digestible carbohydrates commonly used in the non-ruminants for the improvement of gut health and feed utilization. They are also used in rumen manipulation along with nitrates, probiotics, and yeast since they have the potential to reduce CH_4_ production [5]. At present, animal researchers are exploring the efficiency of prebiotic inulin for modulating the gut ecosystem of both ruminants and non-ruminants. In ruminants, the prebiotic reduces rumen ammonia nitrogen, CH_4_ production, increase microbial protein synthesis, and live weight gains in calves [8-10]. On the other hand, probiotics have been defined as living microorganisms that when contained in the feed of animals positively affect the host by improving its digestive system and its weight gain [11,12]. One of the promising probiotics that could improve the health and performance of young calves is the live yeast *Saccharomyces cerevisiae*. Dietary supplementation with *S. cerevisiae* might increase feed intake and energy utilization, strengthen the immune response, and reduce the incidence in diseases of young calves [13]. One potential mode of the action of *S. cerevisiae* is to scavenge oxygen within the rumen creating a more anaerobic environment, which is required by ruminal microorganisms [14]. *S. cerevisiae* is also considered to provide nutrients, such as organic acids, B vitamins, and amino acids, that stimulate microbial growth in the rumen, thereby indirectly stabilizing ruminal pH [15]. Yeast also has the potential to alter the fermentation process in the rumen in a manner that reduces the formation of CH_4_ gas. It has been reported a shift in H_2_ utilization from methanogenesis to reductive acetogenesis by yeast in *in vitro* experiments [6].

As previously described, prebiotic inulin and probiotic *S. cerevisiae* when used alone positively impact animal growth rate and reduce the production of CH_4_, but no research has been conducted so far using these two feed additives together as a synbiotic. This research aimed to measure the amount of CH_4_ and CO_2_ in the rumen and feces after synbiotic addition that contained prebiotic inulin and probiotic *S. cerevisiae*. At the same time, the productivity of calves by comparing the live weight gain and several parameters of meat quality was evaluated.

## Materials and Methods

### Ethical approval

All procedures performed in the present study were in accordance with the ethical standards. Research Committee of the Faculty of Veterinary Medicine, Latvia University of Life Sciences and Technologies approved this study (protocol no. 2017/2).

The method of the collection of rumen fluid was invasive (puncturing the abdomen) and this caused some pain in calves; moreover, some calves were slaughtered at the end of the experiment to obtain samples of meat and gastrointestinal tract for histological examination. Following the ethical requirements to minimize the number of animals used in experiments, we chose to organize as small groups as possible (five animals per group).

### Study period and location

The research was conducted in a dairy farm in Latvia, Saldus District. The research was performed from March until the end of April 2018.

### Animals

Ten clinically healthy randomly selected Holstein Friesian and Red Holstein (*Bos taurus* L.) crossbreed calves with a mean age of 33±6 days and initial body weight of 73.4±12.75 kg were used in the present study. All calves were housed in groups in a partly closed pen in a farm. After birth, all calves received colostrum, and for the next 5 days, calves received whole milk (3.5 L twice a day) and later the milk replacer in a dosage appropriate to their age and weight. Within the age of 4-8 weeks, calves received 8 L of milk replacer per day and a pre-starter diet without restriction (around 0.5 kg/calf/day). After the age of 8 weeks, calves received approximately 1.5 kg of barley flour and 6 L of milk replacer per day. During the experiment, calves had free access to hay and water.

### Experimental design

Calves were allocated into two groups: Five calves in the control group (CoG) receiving a standard diet and five calves that additionally received a synbiotic which consisted of two different products; 12 g of flour of Jerusalem artichoke (*Helianthus tuberosus* L.) per head containing 6 g of prebiotic inulin (produced in Latvia, at the University of Latvia, Institute of Microbiology and Biotechnology) and probiotic 5 g of a yeast culture based on *S. cerevisiae* strain 1026 (Yea-Sacc^®^, Alltech Inc., USA) (SynG). The prebiotic and probiotic were added to barley flour once a day in the morning.

The duration of the experiment was 8 weeks (56 days). We measured the amount of CH_4_ and CO_2_ in the rumen and determined the weight of calves. The full technique is described in our previous study [16]. The samples from calves’ rumen were evaluated 3 times during the research with an interval of 4 weeks – at the 1^st^, 28^th^, and 56^th^ days of the research.

At the end of the experiment, three calves from each group were slaughtered at certified slaughterhouse following all guidelines of humane slaughter. Meat samples from the longissimus muscle were collected and sent to the accredited laboratory for the assessment of some indicators of meat quality (amount of protein [LVS ISO 937:1978] [Kjeldahl method], fat [LVS ISO 1443:1973] [Soxhlet method], [Gravimetry], fatty acids [BIOR-T-012-131-2011] [Gas chromatography], and cholesterol [BIOR-T-012-132-2011] [Gas chromatography]).

Mixed stool samples of each calf group and individual rumen samples of all calves’ group were used to detect methanogens. DNA was isolated by QIAamp^®^ DNA Stool Mini Kit. There was used 200 mg of frozen samples and processed according to the manufacturer’s instructions. DNA amount and purity were verified by NanoDrop-1000, Thermo Fisher Scientific Inc. spectrophotometer. Isolated DNA samples were stored at −20°C till future analyzes.

Specific primer sets were used to detect methanogens. The primer sequences for the methanogens were as follows:

Total methanogens (rrs): Met630F: 5’-GGATTAGATACCCSGGTAGT-3’; Met803R: 5’-GTTGARTCCAATTAAACCGCA-3’.

Total prokaryotes (rrs, reference gene): V3-F: 5’-CCTACGGGAGGCAGCAG-3’; V3-R: 5’-ATTACCGCGGCTGCTGG-3’.

*Methanosphaera stadtmanae* (rrs): Stad-F: 5’-CTTAACTATAAGAATTGCTGG-3’; Stad-R: 5’-TTCGTTACTCACCGTCAAGAT-3’.

*Methanobrevibacter ruminantium* (rrs): Rum16S 740F: 5’-TCCCAGGGTAGAGGTGAAA-3’; Rum16S 862R: 5’CGTCAGAATCGTTCCAGTCA-3’; Rum16S FAM: 5’-CCGTCAGGTTCGTTCCAGTTAG-3’.

*Methanobrevibacter smithii* (rrs): Smit.16S-740F: 5’-CCGGGTATCTAATCCGGTTC-3’; Smit.16S-862R: 5’-TCCCAGGGTAGAGGTGAAA-3’; Smit.16S FAM: 5’CGTCAGAATCGTTCCAGTCA-3’.

Polymerase chain reaction (PCR) was performed using QuantiNova™ Probe PCR Kit and QuantiNova^™^ SYBR^®^ Green PCR Kit following manufacturer’s instructions.

Amplification of DNA was performed in a Rotor-Gene Q real-time PCR cycler using the following conditions: Initial denaturation at 95°C for 2 min followed by 40 cycles of denaturation (95°C for 5 s) and annealing (60°C for 10 s [total methanogens [rrs, mcrA], total prokaryotes, and *M. stadtmanae*] and 60°C for 5 s [*M. ruminantium* and *M. smithii*]).

Methanogen levels were estimated as the value of Ct (PCR cycle number at which sample’s reaction curve intersects the threshold line) was <25 – strong positive, <30 positive, <35 weak positive, and >35 very weak positive.

### Statistical analysis

The assumption of normal data distribution was assessed by Shapiro–Wilk’s test and visual inspection of their histograms and normal Q-Q plots. The assumption of homogeneity of variances was tested by Levene’s test. To determine whether there were any statistically significant differences between three independent groups, we used Kruskal–Wallis H test with pairwise comparisons using Dunn’s procedure [17] with a Bonferroni adjustment. To determine whether there are any statistically significant differences between the two groups, we used the Mann–Whitney U-test or the independent samples t-test. The measure of the strength and direction of the association between two continuous or ordinal variables was evaluated by the Spearman’s rank-order correlation. Those tests were carried out using SPSS Statistics version 22 (IBM Corporation, Chicago, Illinois). All statistical analyses were performed at P=0.05 and data are presented as means ± standard deviation (SD).

## Results

Weight gain values between 1^st^-28^th^, 28^th^-56^th^, and 1^st^-56^th^ days are presented in Table-1. The data of initial and daily live weight gain were normally distributed, and there was the homogeneity of variances. There were no statistically significant differences in mean live weight gain and mean daily weight gain between groups during the 1^st^ month (period 1^st^-28^th^ day) (p>0.05). However, independent samples t-test showed that the weight gain during the whole research was higher in the SynG (62.6±13.75 kg) than CoG (36.8±7.98 kg) calves (p<0.01). The mean daily weight gain was also greater in SynG (1.1±0.24 kg) than CoG (0.7±0.14 kg) calves (p<0.01).

**Table-1:** Effect of synbiotic supplementation on growth performance parameters of calves.

Parameters	Group		p-value

	CoG	SynG	
Initial mean live weight (kg±SD)	79.4±10.52	67.4±12.83	0.145
Mean live weight gain (kg±SD)			
1^st^-28^th^ research days	17.6±4.92	21.8±4.81	0.210
28^th^-56^th^ research days	19.2±4.96	40.8±11.16	0.004*
1^st^-56^th^ research days	36.8±7.98	62.6±13.75	0.007*
Final mean live weight (kg±SD)	116.2±16.30	130.0±25.22	0.334
Mean daily weight gain (kg/day)			
1^st^-28^th^ research days	0.6±0.17	0.8±0.17	0.216
28^th^-56^th^ research days	0.7±0.17	1.5±0.39	0.004*
1^st^-56^th^ research days	0.7±0.14	1.1±0.24	0.007*

* Significant at p<0.05. CoG=Control group, SynG=Synbiotic group, SD=Standard deviation

Meat quality was evaluated as the levels of protein (%) and fat (unsaturated and saturated – %) and cholesterol (mg/100 g). There were no statistically significant differences between all parameters in meat samples obtained from calves from groups CoG and SynG (Table-2).

**Table-2:** Effect of synbiotic supplementation on meat quality traits of calves.

Parameter	CoG	SynG	p-value
Protein (%)	20.1±1.05	20.6±0.80	1.000
Fat (%)	1.0±0.03	1.8±0.85	0.700
Unsaturated	57.5±1.75	52.7±2.73	0.100
Saturated	42.5±1.75	48.1±2.76	0.100
Cholesterol (mg/100 g)	60.1±1.10	56.6±0.15	0.100

*Significant at p<0.05. CoG=Control group, SynG=Synbiotic group

The data of CO_2_ and CH_4_ concentration in calves’ rumen during the 1^st^, 28^th^, and 56^th^ days are presented in Table-3. There were statistically significant differences in the mean amount of CH_4_ on days 28 and 56 of the experiment between the two groups. The higher amounts of CH_4_ were observed in CoG group (p<0.001). The levels of CO_2_ were also significantly higher in CoG in all sampling days.

**Table-3:** Effect of synbiotic supplementation on the mean amount of CH_4_ (mg/m^3^) and CO_2_ (mg/m^3^) in calves’ rumen.

Parameters (mg/m^3^)	Day of experiment	CoG		SynG		p-value
	
		Median	Q1-Q3	Median	Q1-Q3	
CH_4_	1^st^	811.50	107.87-870.45	790.18	442.75-1032.87	0.059
	28^th^	1052.94	983.33-1111.89	659.11	565.04-1015.32	<0.001*
	56^th^	792.06	755.06-873.59	675.41	653.46-700.50	<0.001*
CO_2_	1^st^	3258.54	2864.08-3506.88	2701.65	2419.45-3042.81	<0.001*
	28^th^	4618.15	4378.59-4756.74	4263.82	3553.29-4599.96	<0.001*
	56^th^	4251.28	4045.58-4426.25	3266.07	1358.98-4584.91	0.001*

* Significant at p<0.05. CH_4_=Methane, CO_2_=Carbon dioxide, CoG=Control group, SynG=Synbiotic group, Q1-Q3=Quartile 1- Quartile 3

The mean CH_4_ production in calves’ rumen per kg of body weight in CoG at the end of the experiment was also significantly higher in control than synbiotic group (Me=7.10 mg/m^3^, interquartile range [IQR] 5.61-9.44 vs. Me=5.46 mg/m^3^, IQR 4.60-5.95, respectively, p<0.05). On the other hand, the mean CO_2_ level in calves’ rumen per kg of body weight at the end of the experiment was not significantly different between the two groups (Table-4).

**Table-4:** Effect of synbiotic supplementation on the mean amount of CH_4_ (mg/m^3^) and CO_2_ (mg/m^3^) in calves’ rumen on 1 kg body weight.

Parameters	Day of experiment	CoG		SynG		p-value
	
		Median	Q1-Q3	Median	Q1-Q3	
CH_4/_kg	1^st^	9.63	1.17-9.86	12.95	3.94-16.75	0.421
	28^th^	10.33	9.55-10.90	7.58	5.95-10.88	0.222
	56^th^	7.10	6.35-9.19	5.46	4.60-5.95	0.032
CO_2/_kg	1^st^	38.36	36.32-39.26	44.28	29.47-46.0	0.548
	28^th^	46.42	42.58-48.61	53.96	37.59-54.71	0.841
	56^th^	36.72	34.63-37.07	38.47	9.61-62.95	0.917

*Significant at p<0.05. CH_4_=Methane, CO_2_=Carbon dioxide, CoG=Control group, SynG=Synbiotic group, Q1-Q3=Quartile 1- Quartile 3

No correlation was found between the level of CH_4_, CO_2_ gases, and animal weight on day 1, 28, and 56.

There were no significant differences in the calves’ weight and certain methanogen species in rumen liquid and feces on the 56^th^ day of the experiment. Significantly higher results in the parameter total prokaryotes (V3) (bacteria+archaea) in rumen fluid were in group SynG, whereas significantly higher results in the parameter total methanogens Met630/803 in rumen fluid were in group CoG, p<0.05 (Table-5).

**Table-5:** The mean±standard deviation of PCR cycle number at which the sample’s reaction curve intersects the threshold line in rumen fluid and feces.

Primer sequences	The mean PCR cycle number				

	Rumen fluid (n=5)		p-value	Feces (mixed sample from five samples)	
	
	CoG	SynG		CoG	SynG
Total prokaryotes (V3) (bacteria+archaea)	10.5±0.84	12.3±1.41	0.032*	33.9	13.3
Total methanogens (Met630/803)	31.5±1.75	27.8±2.16	0.049*	23.5	26.6
*M. stadtmanae* Stad.	17.5±1.86	18.1±0.82	0.857	8.8	-
*M. ruminantium* Rum16S	18.3±5.41	22.7±0.71	0.400	17.0	19.4
*Methanobrevibacter smithii* Smit16S	13.4±4.35	17.8±5.82	0.699	10.5	12.7

* Significant at p<0.05. CoG=Control group, SynG=Synbiotic group

## Discussion

Different feed additives such as prebiotics, probiotics, and their combination synbiotics have been used in farm animals to improve their growth performance. Based on our results, we can propose that synbiotic (prebiotic inulin + probiotic *S. cerevisiae* strain 1026) significantly increases live weight gain in calves since increased daily weight gain was observed in SynG calves from the 1^st^ to 56^th^ day of the experiment.

Many studies have proved that different prebiotics and probiotics individually and in various combinations can positively affect the growth rate of different animals. For example, Miguel *et al*. [18] found that inclusion of the prebiotic mannan oligosaccharide (MOS) at different inclusion levels on an as-fed basis (0.1-0.4%) into piglet diet for 14-56 days can improve their growth rate. Similar conclusions were reached by Tang *et al*. [19] that fed piglets for 14 days with dietary supplements of oligosaccharides chitosan 0.025% and galacto-mannan-oligosaccharides 0.20%, and the experimental group showed an improved growth rate.

Several studies show the positive effect of prebiotic inulin on calves’ growth performance. For example, 36 Holstein Friesian breed calves that received inulin from the 1^st^ to the 56^th^ day of life at the dosage 6 g/day/head showed higher final body weight than animals that did not receive inulin [20].

Our results coincide with that of Lesmeister *et al*. [21] and Roodposhti and Dabiri [22]. Lesmeister *et al*. [21] noted an average daily weight gain improvement by 15.6% for 2% yeast (*S. cerevisiae*) treatment in 42 days long experiment, and Roodposhti and Dabiri [22] concluded that the adding of probiotic at 1 g (Protexin^®^; multi-strain probiotic contains 7 bacteria strains and 2 yeast strains with 2×10^9^ CFU/g) and prebiotic at 4 g (a commercial product which contains polysaccharides of *S. cerevisiae* cell wall) per day for 8 weeks (synbiotic) to calves’ feed can significantly improve their average daily weight gain.

Many studies focus on the impact of prebiotics and probiotics on meat quality of different animal species. Most of them showed that prebiotics and probiotics do not affect meat quality. For example, addition of a probiotic *Lactobacillus reuteri* (2.5×10^8^ cfu/mL) to broiler chicken diet over the 42 days of the experiment did not affect meat quality [23]. Another study with inclusion of probiotic *S. cerevisiae* 1 g/kg of diet and prebiotic MOS 1 g per kg of diet into turkey diet for 10 weeks also showed that these feed additives do not influence the different parameters of meat quality, including the amount of protein [24]. Raghebian *et al*. [25] found that probiotic *S. cerevisiae* 3 g and 4.5 g/lamb/day in 84 days long experiment did not significantly impact the amount of fat in lamb meat. Similar results were presented by Gadekar *et al*. [26] who added probiotic *Lactobacillus acidophilus* culture (3.6×10^9^ cells/ml) to lamb diet at dosages 1.0, 1.5, and 2.0 ml/kg body weight for 167 days. Tufarelli *et al*. [27] in a 12-week long study with pigs that were supplemented with a probiotic blend (*Streptococcus thermophilus* DSM 32245, a mixture of two strains *Bifidobacterium animalis* ssp. lactis DSM 32246 and DSM 32247, *L. acidophilus* DSM 32241, *Lactobacillus helveticus* DSM 32242, *Lactobacillus paracasei* DSM 32243, *Lactobacillus plantarum* DSM 32244, and *Lactobacillus brevis* DSM 27961) at the dosage 100 mg/kg of body weight observed that the crude protein content increased significantly in pigs fed a probiotic blend; however, no significant differences were observed on meat crude fat content. In our study, we did not record any statistically significant differences in such meat quality parameters as amount of protein (%) and fat (unsaturated and saturated – %) and cholesterol (mg/100 g).

It has been described that probiotic *S. cerevisiae* can use oxygen within the rumen and by this way, a more anaerobic environment is created, which is required by ruminal microorganisms [14]. Yeast also has the potential to change the fermentation process in the rumen and possibly stimulate the acetogenic bacteria to compete with methanogens or to cometabolize H_2_, thereby reducing the formation of methane gas [6,28,29].

Lynch and Martin [28] in their *in vitro* experiment found a reduction in CH_4_ gas production by 20% after 48 h of incubation of mixed rumen microorganisms containing alfalfa and a live yeast product at concentration of 0.35 and 0.73 g/L. Frumholtz *et al*. [30] also reported outstanding results in another *in vitro* experiment. Authors used the probiotic *Aspergillus oryzae*, and CH_4_ production decreased in the experimental group due to the reduction of the protozoal population (45%). These findings are consistent with those of Hernández *et al*. [31] that have used rumen inoculum of 60 day-old calves supplemented with *S. cerevisiae* (0, 2, and 4 mg/g of dry matter) and incubated it for 70 h. These results support our findings; a significant reduction of the amount of CH_4_ and CO_2_ in calves which received synbiotic consisting of prebiotic inulin and probiotic *S. cerevisiae* was observed.

However, our findings are inconsistent with that of Takahashi *et al*. [32]. They conducted a 4×4 Latin square design experiment with sheep (each test period consisted of 9 days with 7 days for adjustment to feeds) and used a mixture of *Bacillus subtilis*, *Bacillus cereus*, *Bacillus thuringiensis*, *Pseudomonas fluorescens*, *Streptomyces cellulosae*, *Streptomyces albidoflavus*, and *Saccharomyces lipolytica* at the dosage 87 mg/kg body weight and observed an increase in the production of CH_4_ by 18%.

In our previous 56 days long research in which we have used the flour of Jerusalem artichoke at the doses of 12 g (inulin content 6 g) and 24 g (inulin content 12 g), no significant reduction in CH_4_ and CO_2_ gases in calves’ rumen was recorded [16,33]. These results suggest that sole supplementation with the prebiotic inulin does not affect the production of CH_4_ and CO_2_.

Rumen has a high microbial population density comprised of different prokaryotes, eukaryotes, methanogenic archaea, and bacteriophages [34]. The ruminant species have different methanogen populations, for example, *M. ruminantium* and *Methanomicrobium* mobile are major *methanogens* in the ovine rumen [35]. In the cow rumen, Methanobrevibacter seems to be the dominant genus of the archaeal domain [36,37]. In an experiment with feedlot cattle, Wright *et al*. [38] identified following major methanogens in the rumen: *M. ruminantium*, Methanobrevibacter thaueri, *M. smithii*, and *M. stadtmanae*. These findings are consistent with those of Whitford *et al*. [39]; however, *M. ruminantium* were the most abundant rumen methanogen followed by *M. stadtmanae* in that study.

Methanogens produce methane under highly anaerobic conditions [34]. For example, *M. smithii* produce CH_4_ from CO_2_, H_2_, and formate, but *M. stadtmanae* produce methane only through reduction of methanol with H_2_ [40]. In cows’ rumen, certain groups of *Methanobrevibacter* species (*M. smithii*, *Methanobrevibacter gottschalkii*, *Methanobrevibacter millerae*, and *M. thaueri*) are associated with high production of CH_4_ [41-43].

*S. cerevisiae* affects gut microbiota and morphological development in young calves [44]; however, the results of studies on the impact of probiotic *S. cerevisiae* on ruminal CH_4_ production are controversial. Five days long *in vitro* study revealed that *S. cerevisiae* increases the growth of acetogenic bacteria that compete methanogens by utilizing H_2_ and CO_2_ to produce acetate [6]. Ogunade *et al*. [45] stated that yeast *S. cerevisiae* at the dosage 15 g/day might increase the amount of ruminal CH_4_ produced in steers in a 25-day long experiment due to the increased abundance of *M. ruminantium*. Ding *et al*. [46] reported that yeast *S. cerevisiae* could increase rumen bacteria, fungi, and protozoa in steers receiving yeast supplementation (8×10^9^ CFU/h/day through the ruminal fistula) following a two-period crossover design (four phases, each lasted 17 days); also, we recorded that *S. cerevisiae* could increase the number of total prokaryotes (bacteria and archaea). Galindo *et al*. [47] documented the reduction of methanogens and ruminal methanogenesis in 24 h long *in vitro* experiment by adding *S. cerevisiae* on star grass (*Cynodon nlemfuensis* L.) which was used as a substrate to be fermented, a finding that is in contrary to the results of our study. We recorded a significantly higher amount of total methanogens in calves of the CoG. However, separate methanogen species, which are considered to be the primary CH_4_ producers in the rumen (based on information provided before), were in higher amount in synbiotic group. We can assume that the increase of total methanogens in calves from the CoG is due to other species not examined in our study.

## Conclusion

We conclude that synbiotic containing 6 g of prebiotic inulin and 5 g of probiotic *S. cerevisiae* strain 1026, significantly increase the mean daily weight gain in calves. This synbiotic impacts the amount of CH_4_ and CO_2_ gases by substantially decreasing their level in the rumen of calves; however, no correlation was found between these gases and animal weight. Furthermore, the synbiotic does not impact different methanogen species in rumen liquid and feces, and these methanogens do not have any correlation with calves’ weight or amount of produced methane in the rumen. Inulin and yeast *S. cerevisiae* do not have any impact on meat quality parameters, such as protein (%), fat (unsaturated and saturated – %), and cholesterol (mg/100 g) levels. The results of this study showed a significant increase in live weight gain and reduction of GHG emissions in calves; therefore, further research is warranted to elucidate the mechanisms of synbiotic activity.

## Authors’ Contributions

SJ collected the samples, performed the clinical examination of calves, performed the analysis of rumen gases, and drafted the paper and revised it. AI designed the concept for this research and scientific paper. MZ performed the statistical analysis of all data. AB performed the analysis of methanogens. All authors read and approved the final manuscript.
